# Suramin inhibits cell proliferation in ovarian and cervical cancer by downregulating heparanase expression

**DOI:** 10.1186/s12935-015-0196-y

**Published:** 2015-05-13

**Authors:** HuaPing Li, HuaLi Li, HongJie Qu, MingZhu Zhao, Bo Yuan, MingHua Cao, JinQuan Cui

**Affiliations:** Department of Gynaecology and Obstetrics, Punan Hospital of Pudong District, South Pudong Road, Shanghai, China; Department of medicine laboratory, The Food and Drug Administration of pingdingshan, Labor Road, Pingdingshan, China; Department of Gynaecology and Obstetrics, The Second Affiliated Hospital of Zhengzhou University, Longitude eighth Road, Zhengzhou, China

**Keywords:** Suramin, Inhibition, Heparanase, Ovarian cancer, Cervical cancer

## Abstract

**Background:**

Aberrant expression of heparanase (Hpa) is associated with apoor prognosis in ovarian and cervical cancer patients. Inhibitors of Hpa can prevent the growth and metastasis of malignant tumor cells, and suramin may be such a compound that has strong anti-proliferative effects on several kinds of cancer cells. We have therefore tested whether the growth inhibiting effect of suramin on ovarian and cervical cancer cells is due to downregulation of Hpa expression.

**Results:**

Suramin at 300–600 μg/ml significantly inhibited HO-8910 PM and HeLa cell growth at 24 h, in both a time-dependent and dose-dependent manner, with an IC_50_ of 320 μg/ml and 475 μg/ml, respectively. Suramin at 300 μg/ml significantly decreased the expression of Hpa mRNA (*P* < 0.005) and protein (*P* < 0.005) in both HO-8910 PM and HeLa cells at 48 h.

**Conclusions:**

The inhibitory effect of suramin on Hpa enzyme may be due to downregulating of its expression in cancer cells. These findings confirm the importance of Hpa in tumor growth and the potential clinical application of Hpa inhibitors in the treatment of ovarian and cervical cancer.

## Background

Ovarian cancer is the fifth most common cause of cancer death among females and ranks as the first cause of death in gynecologic malignancies. Due to no effective early detection methods, ~70% ovarian cancers are often diagnosed at advanced stage. With the introduction of new treatment modalities, clinical results have been significantly improved, but the ovarian cancer 5-year survival rate is only 44% [1]. Uterine cervical cancer is another major healthcare concern worldwide, especially in the less developed countries. Despite advances in screening, vaccination and treatment of early stage disease, advanced stage tumour, its recurrence and distant metastasis are the main causes of treatment failure and death. Although systemic chemotherapy or radiochemotherapy remains the standard treatment for those patients, their responses to cytotoxic drugs are not satisfactory, and no truly effective second and third line therapeutic regimens are in place. Therefore, new strategies are needed to improve survival and enhance responsiveness to cytotoxic drugs. Molecular target treatment is more effective in most cancers, and is now widely used. Several clinical trials of molecular target treatment have demonstrated their effectiveness in ovarian and cervical cancer [2-7]. But some pitfalls should not be ignored, especially relating to the problem of resistance that is a major challenge facing clinicians. There is the need to develop a second-line treatment strategy for the increasing number of patients who become resistance to the molecular agents.

Hpa is an endo-β-glucuronidase that cleaves heparan sulfate proteoglycans within the extracellular matrix, basement membrane or on the cellular surface, directly or indirectly enhancing cell invasion, migration, and intravasation and extravasation, by releasing various growth factors from heparin-binding [8-10]. Overexpression of Hpa in tumor cells markedly enhances their growth, angiogenesis, and metastasis [11]. Ovarian and cervical carcinomas express higher levels of Hpa mRNA and protein, which is associated with tumor stage at high grade and advanced stage [12-14]. Davidson et al. [15] reported that the expression of Hpa in ovarian cancer is 53% and 90% at the cell membrane and cytoplasm, respectively. Membrane expression in >5% of tumor cells correlates with a shorter overall survival. Immunohistochemical positivity for heparanase was 63.3% (38/60) in cervical cancer patients [14]. Hpa expression is also an independent predictor of poor overall survival, suggesting it is involved in tumor metastasis of female genital tract malignancies [16]. Interestingly, elevated serum Hpa levels correlate with malignant invasion and progression in ovarian cancer [17], which may facilitate disease diagnosis and treatment surveillances. Hpa inhibitor has strong anti-proliferation activity *in vitro* against two human ovarian cell lines, OVSAHO and SKOV-3 [18], and may be one of the potential tumor molecular target therapeutics. A potent Hpa inhibitor, PI-88 (a Phase I/II trials product), is effective in several types of tumor [19,20]. Hpa could lead to a new therapeutic strategy for patients with advanced female genital tract malignancies.

Suramin (8,8′-carbonyl-bis [imino-3,1-phenylenecarbonylimino (4-methyl-3,1- phenylene) carbonylimino] bis-1,3,5-naphthalene-trisulfonic acid) was originally used to treat African parasitic infections, such as Rhodesian and Gambian trypanosomiasis. Due to its anti-proliferative activity against several human tumor cell lines in dose- and time-dependent fashion [21], suramin alone or combined with cytotoxic drugs has been studies in many clinical trials that include ovarian cancer [22,23]. The anti-proliferative mechanism of suramin is still not fully understood, but its activity may be due to it inhibiting the binding of growth factors to their receptors and dissociating receptor-bound growth factors, consequently resulting in loss of signal transduction [24]. Suramin is also considered a potent inhibitor of several nuclear enzymes *in vitro*, including DNA primase, DNA polymerase α, RNA polymerase, DNA topoisomerase II, and reverse transcriptase, which may be important to its cytotoxic activity. Suramin and suramin analogues has also been shown to inhibit Hpa in many human cancer cell line by independent groups [25-28]. Suramin inhibits local tumor invasion and distant metastasis by both a direct and an indirect effect on cell adhesion and migration. New suramin analogues have now been developed to try to improve this antitumor activity and overcome its side-effects [29].

Although suramin significantly inhibits the growth of ovarian cancer and is used in clinical trials, its anti-proliferative effect is not properly understood. Indeed, this lack of knowledge of the drug’s primary mechanism of action has prevented use of suramin in female genital tract malignancies. Accordingly, our study has focused on the *in vitro* cytotoxic activity of suramin against human ovarian and cervical cancer cells. We found that suramin significantly downregulates Hpa expression in its inhibitory effect on the growth of cancer cells.

## Results

### Changes of cell morphology in HO-8910 PM cells and HeLa cells after suramin treatment

**Figure 1:**
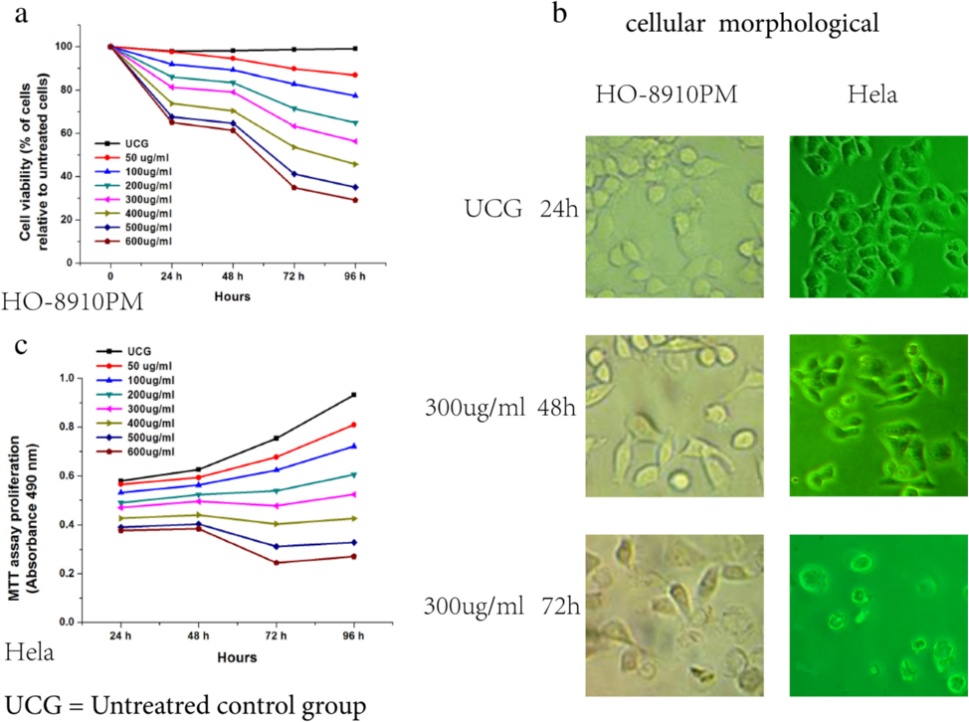
Suramin decreases viability in HO-8910 PM ovarian cancer cells and Hela cervical cancer cells. HO-8910 PM and Hela cells were treated with Hpa inhibitor Suramin (50, 100, 200, 300, 400, 500 and 600 μg/ml). The cells (1 × 10^4^) were incubated at these concentrations for 24, 48, 72 and 96 h at 37°C in a humid air atmosphere containing 5% CO_2_ in RPMI-1640 culture medium. Subsequently, 3-(4,5-dimethylthiazol-2-yl)- 2,5-diphenyltetrazolium bromide (MTT) was added and the cells were incubated for 4 h; viability was assessed by measuring the OD at 490 nm, the value of the untreated control group (UCG) being taken as 100%. The assay showed that the HO-8910 PM and Hela cell vialibility decreased in a dose-dependent and time-dependent manner **(a,c)**. After 48 h treatment with 300 μg/ml suramin, morphological changes became increasingly significant, and viable cells decreased markedly with dead cells floating and clumping in the culture media at 72 h (examined at 4-40X magnification using a Leica DMLB microscope) **(b)**. Statistical analysis, the one-way ANOVA.* P < 0.05 24, 48, 72 and 96 h vs the UCG; 24 vs 72 h, or 96 h; 48 vs 72 h, or 96 h; 72 vs 96 h; ●P < 0.05 100, 200, 300, 400, 500,or 600 μg/ml vs the UCG; 100 vs 300, 400, 500,or 600 μg/ml, 200,vs 300, 400, 500 or 600 μg/ml, 300 vs 400, 500, or 600 μg/ml, 400 vs 500, 600 μg/ml, 500 vs 600 μg/ml.

Changes of cell morphology in HO-8910 PM cells and HeLa cells were explored as part of its dose–response and time–response effects. Clear changes were observed 48 and 96 h post-treatment. Cell density and non-adhesiveness of cells began to decrease and dispersion into single cells increased after 50 μg/ml suramin treatment within 48 h. Membrane blebbing and increased cytoplasmic volume occurred, and viable cells markedly decreased, with dead cells floating and clumping up, in 300 μg/ml suramin within 96 h, suggesting that HO-8910 PM cells and HeLa cells were undergoing apoptosis (Figure 1b).

### Growth changes in HO-8910P and Hela cells after suramin treatment

**Figure 2:**
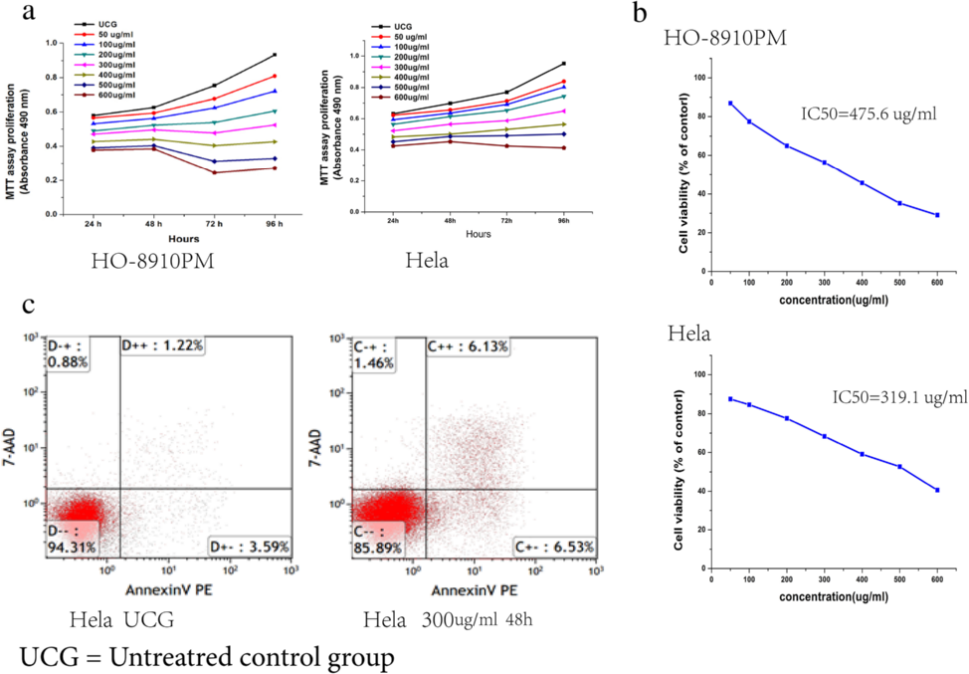
Suramin decreases the proliferation of HO-8910 PM and Hela cells. MTT assay showed that HO-8910 PM and Hela proliferation was inhibited in a dose-dependent and time-dependent manner after suramin treatment **(a)**. IC50 value of HO-8910 PM was 319 μg/mL, IC50 value of Hela was 319.1 μg/mL **(b)**. Flow cytometry was used to detect apoptosis rate in HeLa cells (Figure 2c).The level in cells given 300 μg/ml suramin for 48 h was significantly lower than in untreated cells (300 μg/ml group12.91 ± 1.17%vs UCG 5.01 ± 1.07%,p =0.001) **(c)**.

The growth of the HO-8910 PM and Hela cells using the MTT assay showed that different doses of suramin significantly inhibited growth rate from 24 to 96 (Figure 2a). Inhibition with 600 μg/ml suramin at 96 h reached 70.9% in HO-8910 PM cells and 59.5% in Hela cells. Except for the 50 μ g/ml group vs 100 μ g/ml group, inhibition of the other groups of HO-8910 PM cells showed significant differences (Ftime = 38.128, Ptime = 0.0001,Fdose = 44.984, Pdose = 0.0001). For HeLa cells, except for 50 μg/ml group vs 100 μg/ml, and vs 200 μg/ml group, inhibition of the other groups was significantly different (Ftime = 20.548, Ptime = 0.0001,Fdose = 32.324, Pdose = 0.0001). The IC50 values of HO-8910 PM and HeLa were 319 μg/ml, 476 μg/ml, respectively (Figure 2b).Plasma concentration of ≥350 μg/ml suramin led to a dose-limiting neurotoxicity [30] . At 96 h, treatment with 200 and 300 μg/ml suramin inhibited 35.1- 43.7% of HO-8910 PM cell growth and 22.4-31.7% of Hela cell growth, confirming the toxic nature of suramin. Flow cytometry was used to detect apoptosis rate in HeLa cells (Figure 2c).The level in cells given 300 μg/ml suramin for 48 h was significantly lower than in untreated cells (300 μg/ml group12.91 ± 1.17%vs UCG 5.01 ± 1.07%,p =0.001).

### Suramin inhibits HO-8910 PM and Hela cell proliferation

**Figure 3:**
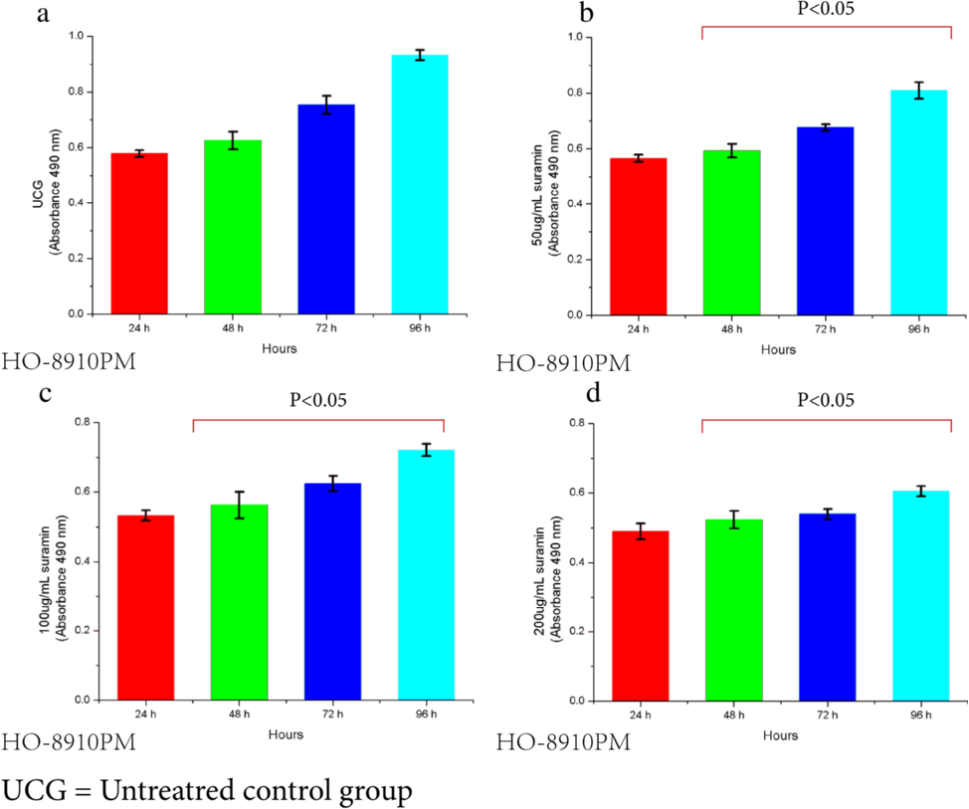
Viability decrease in HO-8910 PM treated with 0, 50, 100 and 200 ug/ml Suramin. Suramin decreased viability and proliferation of HO-8910 PM cells at 50, 100, 200, 300, 400, 500 and 600 μg/ml **(a,b,c,d)**. HO-8910 PM (1 × 10^4^) were incubated in the presence of these concentrations for 24, 48, 72 and 96 h at 37°C in a humid atmosphere containing 5% CO_2_ and 95% air in RPMI-1640 culture medium. MTT was added and the cells were incubated for 4 h; viability was estimated by measuring the Optical density (OD) at 490 nm. The results represent the mean ± the standard deviation (SD) of all independent experiments performed in triplicate. Statistical analysis by one-way ANOVA. * P < 0.05 50 μg/ml 48 h vs 50 μg/ml 72 h; 50 μg/ml 72 h vs 50 μg/ml 96 h **(b)**. ●*P* < 0.05: 100 μg/ml 48 h vs 100 μg/ml 72 h; 100 μg/ml 72 h vs 100 μg/ml 96 h **(c)**. ♦ *P* < 0.05: 200 μg/ml 48 h vs 200 μg/ml 72 h; 200 μg/ml 72 h vs 200 μg/ml 96 h **(d)**.

**Figure 4:**
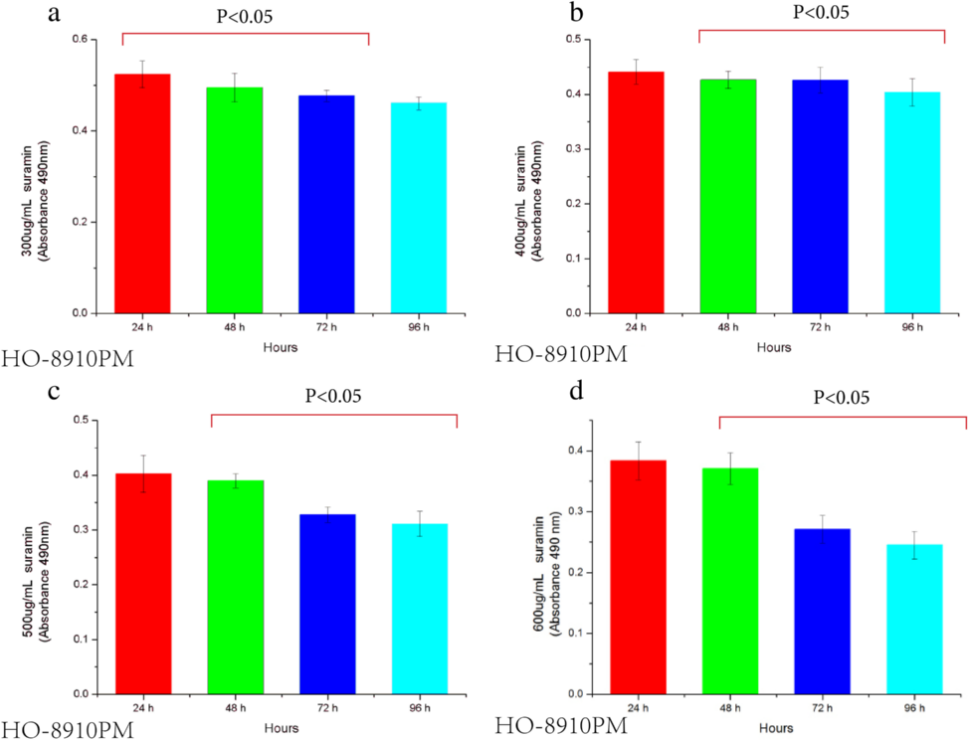
Viability decrease of HO-8910 PM cells treated with 300, 400, 500 and 600ug/ml Suramin **(a,b,c,d)**. The results represent the mean ± the standard deviation (SD) of all independent experiments performed in triplicate. Statistical analysis, the one-way ANOVA. * P < 0.05 300 μg/ml 48 h vs 300 μg/ml 72 h; 300 μg/ml 72 h vs 300 μg/ml 96 h **(a)**. ●*P* < 0.05 400 μg/ml 48 h vs 400 μg/ml 72 h; 400 μg/ml 72 h vs 400 μg/ml 96 h **(b)**. ♦ *P* < 0.05 500 μg/ml 48 h vs 500 μg/ml 72 h; 500 μg/ml 72 h vs 500 μg/ml 96 h **(c)**. ◎*P* < 0.05 600 μg/ml 48 h vs 600 μg/ml 72 h; 600 μg/ml 72 h vs 600 μg/ml 96 h **(d)**.

**Figure 5:**
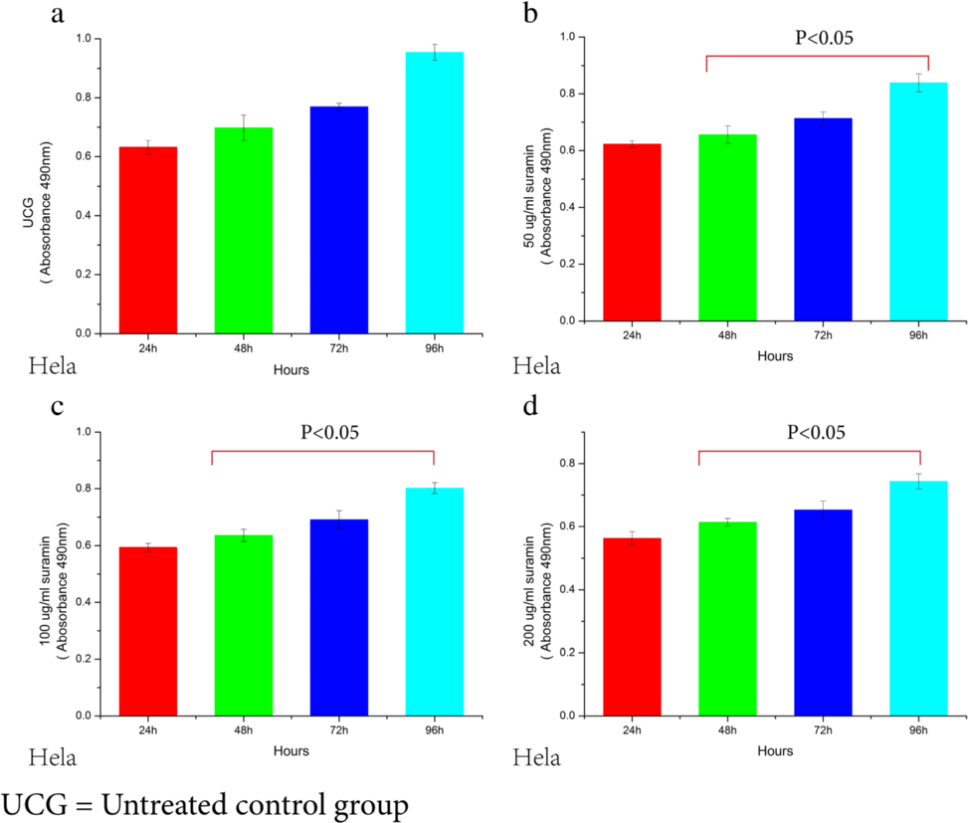
Viability decrease in Hela cells treated with 0, 50, 100 and 200ug/ml Suramin **(a,b,c,d)**. The results represent the mean ± the Standard deviation (SD) of all independent experiments performed in triplicate. Statistical analysis, the one-way ANOVA. * P < 0.0 50 μg/ml 48 h vs 50 μg/ml 72 h; 50 μg/ml 72hs vs 50 μg/ml 96 h **(b)**. ●*P* < 0.05 100 μg/ml 48hs vs 100 μg/ml ;100 μg/ml 72 h vs 100 μg/ml 96 h **(c)**. ♦ *P* < 0.05 200 μg/ml 48 h vs 200 μg/ml 72 h; 200 μg/ml 72 h vs 200 μg/ml 96 h **(d)**.

**Figure 6:**
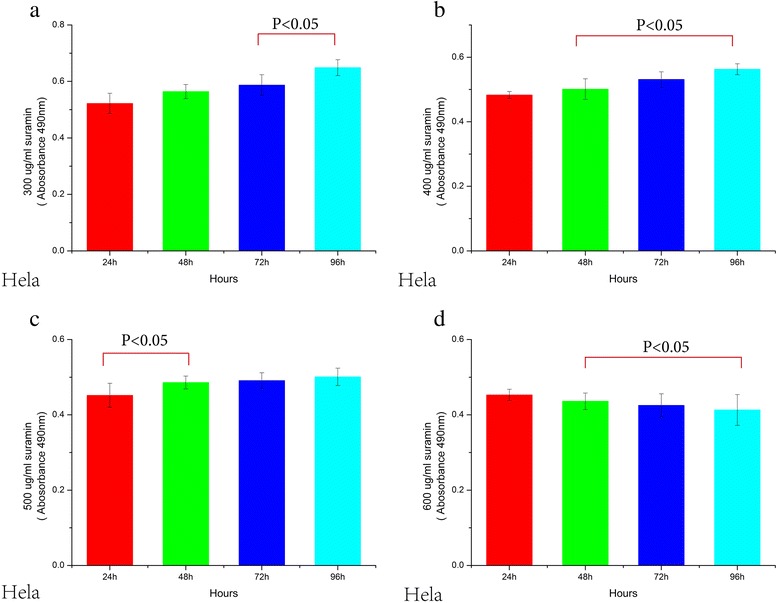
Viability decrease in the Hela cells treated with 300, 400, 500 and 600ug/ml Suramin **(a,b,c,d)**. The results represent the mean ± the Standard deviation (SD) of all independent experiments performed in triplicate. Statistical analysis, the one-way ANOVA. * P < 0.05 300 μg/ml 72 h vs 300 μg/ml 96 h **(a)**. ●*P* < 0.05 400 μg/ml 48 h vs 400 μg/ml 72 h;400 μg/ml 72 h vs 400 μg/ml 96 h **(b)**. ♦ *P* < 0.05 500 μg/ml 24 h vs 500 μg/ml 48 h **(c)**. ◎*P* < 0.05 600 μg/ml 48 h vs 600 μg/ml 72 h; 600 μg/ml 72 h vs 600 μg/ml 96 h **(d)**.

Proliferation of HO-8910 PM and HeLa cells treated with suramin showed time-dependency and dose–dependency. With increasing of dose and time, proliferation gradually decreased until 96 h. OD values of different groups (24, 48, 72 and 96 h) and 7 different doses(50,100,200,300,400,500,600 μg/ml)were significantly lower than the untreated controls (UCG) (F_time_ = 480, P_time_ = 0.0001, F_dose_ = 1655, P_dose_ = 0.0001 for HO-8910 PM; F_time_ = 126, P = 0.0001; F_dose_ = 768, P_dose_ = 0.0001 for HeLa). For statistical analysis of inter-group OD values in both HO-8910 PM and HeLa cells, all p values were equal to 0.0001 (0.000 is not a value) either in 4 different time groups or in 7 different dose groups, except for the 24 h vs 48 h groups ( Figures 3, 4, 5 and 6).

### Suramin downregulates the expression of Hpa protein and mRNA in HO-8910 PM and Hela cells

**Figure 7:**
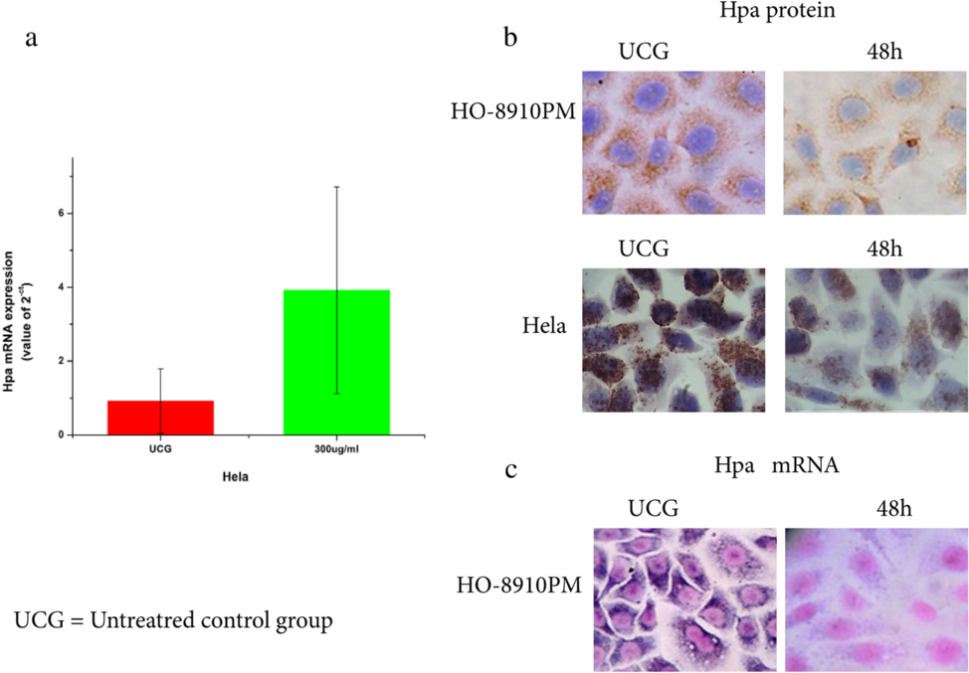
Effect of suramin on Hpa protein and mRNA expression HO-8910 PM and the Hela cells. Slides of 48 h post-treatment with 300 μg/ml were selected for study. Both groups were tested in sextuplicate, and the experiment was repeated 3 times. Expression of Hpa protein was followed by SP immunohistochemical staining. Sections of colon cancer tissues positive for Hpa protein expression were used as the positive control, while antibody diluent replaced the antibody in the negative control. Compact brown or yellow immune particles of Hpa protein were present in the cytoplasm in the UCG, which distributed diffusely in all fields. The staining signals of Hpa protein in 300 μg/ml group decreased significantly in intensity as well as integration seen with a zoom lens of 40X by microscopy **(b)**. HO-8910 PM cell Hpa mRNA expression was measured by an *in situ* hybridization kit. The slides were processed with an excessive amount RNA enzyme as the negative control. Sections of colon cancer tissues were used as the positive control. Compact blue hybridized granules of Hpa mRNA were present in the cytoplasm of the UCG, which distributed diffusely in all fields. The staining signals of Hpa mRNA in the 300 μg/ml group decreased significantly in intensity as well as integration **(c)**. Real-time quantitative-PCR was used to determine Hpa mRNA expression in HeLa cells **(a)**; its level in cells treated with 300 μg/ml suramin for 48 h was significantly lower than in untreated cells (300 μg/ml group 0.92 **±** 0.87vs UCG 3.62 **±** 2.80). Statistical analysis, chi-square test; *P <0.05. Hpa protein expression of 48 h post-treatment in the 300 μg/ml group *vs* the UCG; ●*P* < 0.05. Hpa mRNA expression of 48 h post-treatment in the 300 μg/ml group *vs* UCG.

Suramin downregulation of the expression of Hpa protein in HO-8910 PM and Hela cells was investigated by immunocytochemistry. From the MTT results, we selected 300 μg/ml suramin and 48 h treatment as appropriate (Figure 7b). Compact brown or yellow immune particles of Hpa protein and blue hybridized granules of Hpa mRNA were present in the cytoplasm in the experimental control group, distributed diffusely in all fields (Figure 7c). Staining in the suramin group decreased significantly in intensity as well as integration (χ2 = 25.958, P = 0.0001,χ2 = 27.091, P = 0.0001). Real-time quantitative-PCR was used to determine Hpa mRNA expression in HeLa cells (Figure 3a). The level in cells given 300 μg/ml suramin for 48 h was significantly lower than in untreated cells (300 μg/ml group 0.92 ± 0.87vs UCG 3.62 ± 2.80, p <0.05), with down-regulation being 3.93 fold lower.

## Discussion

Suramin treatment significantly decreases cell growth in a dose- and time-dependent manner in HO-8910 PM and HeLa cells, consistent with previous reports [21,31,32]. Although the IC_50_ of suramin against in HO-8910 PM cell was 10 times higher compared with other studies, it was lower than the cytotoxic concentrations >275 μM in human blood serum [22]. Thus suramin not only downregulates Hpa protein and mRNA expression, but inhibits the growth of cancer cells.

Suramin is a multiple sulfonic acid naphthoquinone salt that has anti-proliferative activity against many cancers, with a wide range of IC_50_ values. Suramin at 275 μM sustained over several weeks has been associated with severe multitoxicity, including renal toxicity, adrenal insufficiency, immune- and anticoagulant-mediated blood dyscrasias, and dermatological toxicity [33-35]. We found the IC_50_ of suramin to be 319 μg/ml, and at 200 and 100 μg/ml it could inhibit cell growth by 35.1 to 43.7%, while at 210 μg/ml it effectively decreased CA125 serum levels of the patients with ovarian cancer. Non-cytotoxic doses of suramin ranging between 10 and 50 μM enhances the antitumor effects of several chemotherapeutic agents in a number of tumor cell lines [36] and several animal models [37]. Despite the U.S. Food and Drug Administration disapproving the use of suramin at therapeutic concentrations, 2 clinical trials at non-cytotoxic suramin levels in combination with chemotherapeutic agents have been conducted to treat metastatic breast cancer [38] and advanced non-small cell lung cancer [22], with discernible antitumor activity being noted in the latter. Thus, low or non-cytotoxic doses of suramin might be used as effective chemosensitizers to cytotoxic drugs, ie as an adjuvant, for ovarian cancer in the future.

Inhibiting the activity of key enzymes is an important anti-proliferative mechanism of suramin at therapeutic. A growing body of evidence has shown that suramin is the inhibitor of such enzymes as histone methyltransferases [39], histone deacetylases [40], ATPase [41], DNA topoisomerase [42] and Hpa [28]. However, Hpa has been the target molecule here because it is important in tumor cell metastasis through the degradation of heparan sulfate proteoglycans in the extracellular matrix. The human Hpa gene is located on chromosome 4q21.3 with a full-length cDNA of 1758 bp and its active protein product is 50kD. Because of the inherent role of Hpa, inhibition of its activity makes it a potential target in anti-cancer therapy. Numerous Hpa inhibitors are being developed and tested for growth inhibition of tumors. PI88, one of the inhibitors, has been already used in clinical trials.

New suramin analogues have been synthesized possessing highly anti-proliferative effects on tumor cells *in vitro* and angiostatic effects compared with suramin itself. Marchetti et al. [28] reported that the IC50 of suramin on Hpa activity in melanoma 70 W cells was 42 μM, much higher than the IC50 values of suramin analogues (NF 127, NF 145 and NF 171) in a range of 20–30 μM. They also found that Hpa expressed in 70 W cells was effectively inhibited by suramin analogues in a dose-dependent manner. Complete inhibition was obtained at 100 μM and higher. In our study, HO-8910 PM with its high expression of Hpa protein and mRNA comes from a peritoneal metastasis of epithelial ovarian cancer. Treatment with 300 μg/ml suramin significantly decreased Hpa mRNA and protein expression at 48 h, which not only support previous studies, but extend our understanding of the antiprolierative mechanism of suramin on ovarian cancer.

Zeng et al. [14] found 63.3% cervical cancers expressed Hpa, and overexpression of heparanase inhibited apoptosis of cervical cancer cells. Ectopic heparanase overexpression can promote proliferation of cervical cancers *in vitro* and tumor growth *in vivo*. High risk human papillomavirus (HR-HPV) infection has been considered a primary cause of cervical cancer because it is present in the 99.7% of cervical cancers [43,44] , which leads to #0.5 million cases per year. HPV 16 and 18 are the most common HR-HPV types worldwide and account for ~70% of cervical squamous cell carcinomas and up to 85% of the adenocarcinomas. HPV16 E6 and HPV 18 E7 are best known for their ability to target the 2 tumor suppressors, p53 and pRb [45]. The HPV16 oncogene E6 is capable of inducing overexpression of heparanase in head and neck squamous cell carcinoma *in vitro*, which can be suppressed by radiation in a dose-dependent manner. The heparanase gene is regulated through wild-type p53 binding to the heparanase promoter [46]. E6-mediated degradation served as the major mechanism inactivating p53 [47] that might lead to induction of heparanase expression and promote cervical carcinogenesis. Therefore, this study provides important experimental evidence of Hpa-targeted therapy for cervical cancer. Our investigations will now focus on HPV E6 inhibition by suramin.

In conclusion, suramin inhibits strongly the growth of human ovarian and cervical cancer cells, and at same time significantly downregulates Hpa expression. We suggest that the antitumor activity of suramin may partly relate to inhibition of Hpa expression in cancer cells. Therefore, combination of low dose suramin and chemotherapeutic agents for the advanced female genital tract malignancies might be a suitable means of treatment in the future.

## Conclusions

Suramin as a Hpa inhibitor can inhibit the growth of HO-8910 PM and HeLa cells and at same time significantly downregulates Hpa expression.

## Methods

### Cell lines and reagents

Human ovarian cancer cell line, HO-8910 PM, classified as a highly malignant peritoneal metastatic carcinoma with a highly metastatic potential, was obtained from Shanghai institutes for biological sciences (Shanghai, China). Human Cervical cancer line Hela was also obtained from the same source. Suramin as its sodium salt was purchased from EMD Millipore (Billerica, MA, USA). Hpa antibody was purchased from Santa Cruz Biotechnology (Santa Cruz, CA, USA). SP immunohistochemical and DAB kits were purchased from the Beijing Sequoia (Beijing, China). The biotin-labeled Hpa probe was obtained from Beijing Aoke biotechnology company (Beijing, China), and the mRNA sequence was 5′TCA ATG GTG ACG GAC AGG AAC GAG G 3′. *In situ* hybridization reagent box was the production of Boster (Wuhan, China).

### Culture

HO-8910 PM and Hela cells were cultured in RPMI-1640 (Logan, UT, USA) supplemented with 10% fetal bovine serum and incubated at 37°C in a humidified incubator with air plus 5% CO_2_. The cells at 80% confluence in logarithmic growth phase were used for experiments. Viability was >95%, as estimated by trypan blue staining.

### Preparation of adherent cell slides

HO-8910 PM and Hela cells in logarithmic growth phase were detached by digesting with 0.25% trypsin plus 0.02% EDTA. One × 10^4^ cells in 1 ml RPMI-1640 medium supplemented with 10% FBS were seeded on to coverslips pre-placed in welsl in a 24-well plate. After attachment of 70-80%, the medium was poured off and the layer washed twice with ice-cold PBS. The coverlips were removed and transferred to new fresh 24-well plates. They were incubated in 4% paraformaldehyde at 4°C for 30 min and washed twice with PBS before being kept at 20°C for a week.

### MTT assay

Cells were seeded in culture medium in a 96-well plate at 1 × 10^4^ cells/well. After overnight incubation, cells were treated with different concentrations of suramin. MTT (3-(4,5-Dimethylthiazol-2-yl)-2,5-diphenyltetrazolium bromide) assay was used to measure tumor cell viability at from after 24 to 96 h of incubation. The cells were then stained with MTT (Amresco, Solon, OH, USA) for 4 h. The medium was discarded, and the cells were solubilized with 150 μl DMSO for 15 min, and the plate shaken gently for 10–15 min. The wells without suramin treatment were used as the untreated control groups, and sextuplicate wells were tested for each concentration. Each experiment was repeated 3 times. Photometric value A (OD value) at 490 nm was measured by a microplate reader. Growth inhibition rate (GI) was calculated according to the following formula:

$$ \mathrm{G}\mathrm{I}\%=\left[1-\mathrm{A}490\left(\mathrm{experimental}\;\mathrm{group}\right)/\mathrm{A}490\left(\mathrm{untreated}\;\mathrm{control}\;\mathrm{group}\right)\times 100\right]. $$

### Immunocytochemistry

Hpa expression was examined by the SP immunohistochemical method. Briefly, after treated with 3% hydrogen peroxide for 20 min, cell slides were incubated overnight at 4°C with primary anti-Hpa antibody (1: 100). DAB (Diaminobenzidine) staining was given for 10 min. Following hematoxylin counterstaining, the slides were sealed with neutral gum. Sections of colon cancer tissues positive for Hpa expression were used as control, while antibody diluent replaced the Hpa antibody as the negative control. The slides of suramin treatment were defined as the experiment group, and slides without suramin were the untreated controls. Both groups were tested in sextuplicate, and the experiment was repeated 3 times.

### *In situ* hybridization

The equipment and buffers were treated for the hybridization test with diethylpyrocarbonate (DEPC, Sigma-Aldrich, St Louis, MO, USA). After being washed with 50 and 30% alcohol, sterile water and PBS at room temperature, the cells on the slides were permeated in 0.3% Triton X-100/PBS for 10 min and digested with 3% pepsin diluted in fresh citric acid for 20 min at 37°C. After being washed in PBS for 5 min, the slides were post-fixed with 4% paraformaldehyde for 10 min at room temperature. Each slide was dripped with 20 μl hybridization solution and pre-hybridized in the wet box at 37°C for 3-4 h. Hpa probe (6–12 ng/20 μl) was added to each slide. The slides were covered with wax membrane and hybridized for 12 ~ 16 h at 42 ~ 43°C, The slides were washed followed by washing four times in 0.2× SSC at 42°C for 15 min. They were then incubated in 1% acetylated BSA at 20°C for 10 min and in freshly diluted buffer SA-AP (Promega, Madison, WI) for 20 min at 37°C. After washing with Tris–HCl buffer I 3 times and in Tris–HCl buffer II twice, the slides were protected from light and colored in freshly prepared BCIP/NBT (Promega) for 100–120 min. They were then stained with nuclear fast red for several min and sealed with neutral gum. The negative experimental control slides were processed with an excess of RNA enzyme. Sections of colon cancer tissues with positive expression of Hpa were used as positive experimental controls. Both groups were tested in sextuplicate, and the experiment was repeated 3 times.

### Interpretation of immunocytochemistry and *in situ* hybridization results

Positive expression of Hpa protein was determined by yellow, brown and tan particles in the cytoplasm (Figures 2A and B). Hpa mRNA was colored by NBT/BCIP, as shown in blue and purple granules. Positive signals were located in the cytoplasm (Figures 2C and D). Five high-perspectives under binocular microscope were selected in each slide, and 100 cells were counted randomly per field. Expression of Hpa protein and mRNA were scored based on integrated staining intensity and by the proportion of positive cells [48]. The percentage of positively stained cells was scored as 1 (<5%, negative), 2 (5-25%, sporadic), 3 (25-50%, focal) or 4 (>50%, diffuse). Intensity of the hybridization signal was divided into 4 grades: no staining (1 score), weak (2 score), moderate (3 score) and strong (4 score). According to the product of the scores of positive cells and the scores of staining intensity, the expression level was defined as follows: 1+ (<4 scores), ++ (4–8 scores), +++ (9–12 scores), ++++ (13–16 scores).

### TaqMan real-time qRT–PCR

For TaqMan real-time qRT–PCRs, Hpa and b2-microglobulin were purchased from Ambion (Carlsbad, CA, USA; ID: Hs00935033_m1, and 4333766 F, respectively). PCRs were done with the ABI 7500 Fast Real Time PCR System (Applied Biosystems, Foster City, CA, USA). Amplification conditions were 2 min at 50°C, 10 min at 95°C and then 40 cycles each consisting of 15 s at 95°C and 1 min at 60°C. Hpa relative expression was assessed using the comparative C_T_ method and presented as 2^-Δct^ value. The fold changes of Hpa expression were calculated according to reference [49].

### Statistical methods

SPSS10.0 software package (SPSS Inc., Chicago, IL, USA) was used for statistical analysis and calculating the IC_50_ of suramin. The values represent the mean ± the SD of the values obtained. In some experiments, the GI% was calculated, which represents the percentage increase or decrease in relation to the untreated control group. One way ANOVA was used to make comparisons between the groups. For the count data, the Chi-square test was used to calculate the significance of differences between the groups, with *p* < 0.05 being taken as significant.
